# Hyperphosphatemia induces senescence in human endothelial cells by increasing endothelin‐1 production

**DOI:** 10.1111/acel.12664

**Published:** 2017-08-31

**Authors:** Gemma Olmos, Patricia Martínez‐Miguel, Elena Alcalde‐Estevez, Diana Medrano, Patricia Sosa, Leocadio Rodríguez‐Mañas, Manuel Naves‐Diaz, Diego Rodríguez‐Puyol, María Piedad Ruiz‐Torres, Susana López‐Ongil

**Affiliations:** ^1^ System Biology Department Alcala University Alcalá de Henares Madrid Spain; ^2^ Instituto Reina Sofía de Investigación Nefrológica IRSIN Madrid Spain; ^3^ Research Unit Biomedical Research Foundation from Príncipe de Asturias University Hospital Alcalá de Henares Madrid Spain; ^4^ Nephrology Section Biomedical Research Foundation from Príncipe de Asturias University Hospital Alcalá de Henares Madrid Spain; ^5^ Geriatric and Frailty Section Getafe University Hospital Getafe Madrid Spain; ^6^ Bone and Mineral Research Unit Asturias Central University Hospital Oviedo Spain

**Keywords:** AP‐1, endothelin‐1, endothelial cells, hyperphosphatemia, reactive oxygen species, senescence

## Abstract

Hyperphosphatemia is related to some pathologies, affecting vascular cell behavior. This work analyzes whether high concentration of extracellular phosphate induces endothelial senescence through up‐regulation of endothelin‐1 (ET‐1), exploring the mechanisms involved. The phosphate donor β‐glycerophosphate (BGP) in human endothelial cells increased ET‐1 production, endothelin‐converting enzyme‐1 (ECE‐1) protein, and mRNA expression, which depend on the AP‐1 activation through ROS production. In parallel, BGP also induced endothelial senescence by increasing p16 expression and the senescence‐associated β‐galactosidase (SA‐ß‐GAL) activity. ET‐1 itself was able to induce endothelial senescence, increasing p16 expression and SA‐ß‐GAL activity. In addition, senescence induced by BGP was blocked when different ET‐1 system antagonists were used. BGP increased ROS production at short times, and the presence of antioxidants prevented the effect of BGP on AP1 activation, ECE‐1 expression, and endothelial senescence. These findings were confirmed *in vivo* with two animal models in which phosphate serum levels were increased: seven/eight nephrectomized rats as chronic kidney disease models fed on a high phosphate diet and aged mice. Both models showed hyperphosphatemia, higher levels of ET‐1, and up‐regulation in aortic ECE‐1, suggesting a direct relationship between hyperphosphatemia and ET‐1. Present results point to a new and relevant role of hyperphosphatemia on the regulation of ET‐1 system and senescence induction at endothelial level, both in endothelial cells and aorta from two animal models. The mechanism involved showed a higher ROS production, which probably activates AP‐1 transcription factor and, as a result, ECE‐1 expression, increasing ET‐1 synthesis, which in consequence induces endothelial senescence.

## Introduction

Hyperphosphatemia is a pathological condition related to chronic kidney disease (CKD) and more recently found on premature aging syndromes (Razzaque *et al*., 2006; Nakatani *et al*., 2009; Ohnishi & Razzaque, 2010). High concentration of serum phosphate has profound effects on vascular cell behavior and on vascular function, and has been associated with cardiovascular disease in patients with CKD. Phosphate toxicity has been related with many other organ dysfunctions revised by Razzaque (Ohnishi & Razzaque, 2010; Razzaque, 2011), such as impaired fertility, increased lung tumorigenesis, and increased cell death. However, less is known about the effect of hyperphosphatemia on endothelial cells. A few works have described that a high extracellular phosphate level induces endothelial dysfunction via various mechanisms, including a decline in nitric oxide (NO) release due to oxidative stress, associated with reduced intracellular calcium, increased protein kinase C‐β_2_, reduced cell viability, and increased apoptosis (Peng *et al*., 2011); or via inhibitory phosphorylation of endothelial nitric oxide synthase (Shuto *et al*., 2009). Besides, Van *et al*. demonstrated that dietary phosphate restriction ameliorated not only hyperphosphatemia but also the impaired vasodilation of aorta (Van *et al*., 2012), according with the findings of Shuto *et al*. (Shuto *et al*., 2009), who found that dietary phosphorus acutely impairs endothelial function. Only one work refers to endothelin‐1 (ET‐1), in which sevelamer, a phosphate binder, decreased not only phosphate levels but also serum ET‐1, plasminogen activator inhibitor‐1, C‐reactive protein, and interleukin‐6, improving the endothelial function in patients on peritoneal dialysis (Chennasamudram *et al*., 2013).

Endothelium exerts multiple functions to preserve vascular homeostasis. Vasoactive endothelial factors such as NO or ET‐1 are involved in this regulation. An unbalanced production of these bioactive mediators results in endothelial dysfunction, a critical event in the development of renal and cardiovascular damage in some diseases such as diabetes, hypertension, or atherosclerosis (Ludmer *et al*., 1986; Calver, Collier, Moncada, Vallance 1992; Calver, Collier, Vallance 1992Katz *et al*., 1992). On the other hand, vascular dysfunction has been related to endothelial senescence (Regina *et al*., 2016). Cellular senescence was discovered by Hayflick and Moorhead (Hayflick & Moorhead, 1961). It is considered one of the hallmarks of aging (Collado *et al*., 2007), and the presence of senescent cells in the tissue can induce or increase some pathologies (Burton & Krizhanovsky, 2014). Senescent cells are not able to proliferate and present some morphological and biochemical changes, such as increased senescent activity of β‐galactosidase (SA‐β‐GAL) due to an increase in lysosomal content (Dimri *et al*., 1995), and increased expression of cell cycle inhibitors such as p16 or p53 tumor suppressor genes.

Senescence can be promoted by the replicative life of cells, due to the progressive telomere shortening (Collado *et al*., 2007) or prematurely in response to stressful stimuli, which result in DNA damage (Wang *et al*., 2009) or oncogene activation (Serrano *et al*., 1997). Recent studies from our group have demonstrated that hyperphosphatemia could be one of these stressful stimuli, as it induced cellular senescence in human aortic smooth muscle cells through the activation of IGF‐1 receptor and integrin‐linked kinase overexpression (Troyano *et al*., 2015).

The generation of ROS plays a relevant role in the intracellular mechanisms involved in the induction of cellular senescence. Oxidative stress is involved in many diseases, such as cancer and inflammation, and it plays a relevant role in aging (Chen & Ames, 1994; Radisky *et al*., 2005; Lu & Finkel, 2008). Thus, the use of antioxidant compounds can prevent or delay cellular senescence (Macip *et al*., 2002). It is also well known that oxidative stress can modify the endothelium function (Regina *et al*., 2016).

The present work shows, for the first time, the key role of ET‐1 in the senescence process induced by hyperphosphatemia. We show that a high extracellular phosphate concentration up‐regulates the synthesis of ET‐1 in endothelial cells, inducing cellular senescence through the modulation of ECE‐1 via ROS production and AP‐1 activation.

## Results

### Hyperphosphatemia induces ET‐1 synthesis and ECE‐1 expression through AP‐1 activation in cultured human endothelial cells

**Figure 1:**
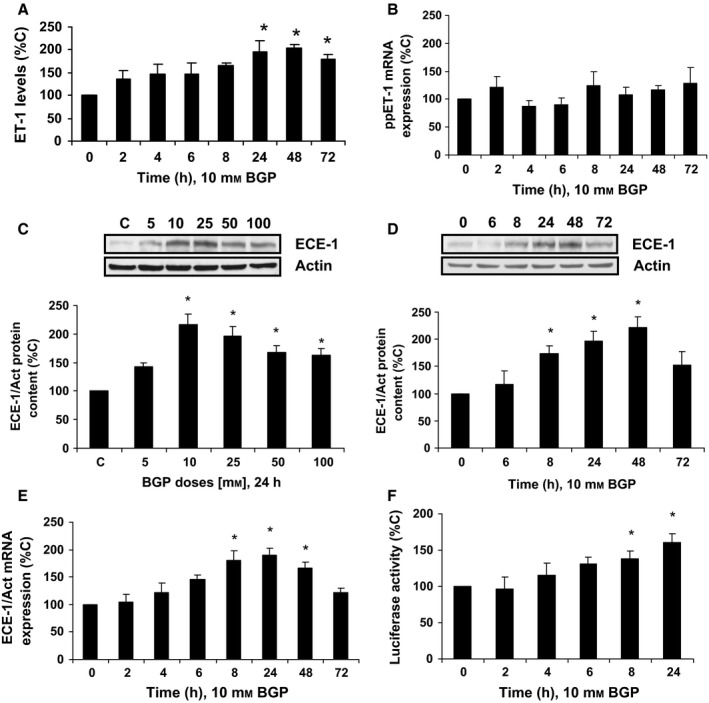
Hyperphosphatemia modulates the endothelin system in human endothelial cells. Cells were incubated with β‐glycerophosphate (BGP) at different times (10 mm) or different doses (24 h); then, ET‐1 synthesis (A), prepro‐ET‐1 mRNA expression (B), ECE‐1 protein content (C and D), ECE‐1 mRNA expression (E), and ECE‐1 promoter activity (F) were measured. In the experiments of the analysis of protein content, a representative Western blot is shown at the top with the densitometric analysis below (C,D). Values are the mean ± SEM of six independent experiments, *P < 0.05 vs. control cells (C or time zero). In all panels, statistical test applied was one‐way ANOVA with repeated measures, followed by Dunnett's multiple comparison test.

Beta‐glycerophosphate (BGP) was used as a phosphate donor to treat human endothelial cells at different doses and times. BGP was able to induce ET‐1 production (Fig.  1A) reaching a maximum effect at 24 h; however, prepro‐ET‐1 mRNA expression was not modified (Fig.  1B). Then, ECE‐1 expression was tested by Western blot and qPCR. ECE‐1 protein content increased in a dose and time manner (Fig.  1C and D), as well as ECE‐1 mRNA expression (Fig.  1E). The increase in ECE‐1 mRNA expression was due to transcriptional changes as BGP raised also ECE‐1 promoter activity (Fig.  1F).

**Figure 2:**
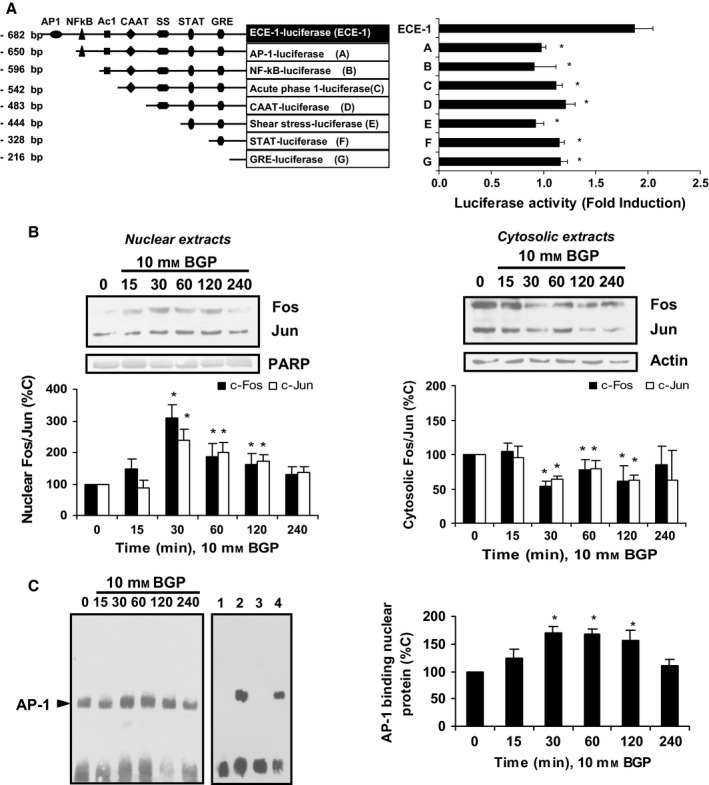
Hyperphosphatemia induces transcriptional modulation of endothelin‐converting enzyme‐1 through AP‐1 activation in human endothelial cells. (A) Endothelial cells were transfected with a plasmid linked to luciferase containing the complete human ECE‐1 promoter (ECE‐1) or serial deletion fragments (A to G) as indicated in A on the left; after that, 10 mm of β‐glycerophosphate (BGP) was added for 24 h**,** and then, luciferase activity was assessed. Phorbol myristate acetate (3 × 10^−7^ m, 6 h) was used as positive control (235% vs. C). (B) Cells were incubated with BGP at different times. c‐Fos and c‐Jun protein expressions were analyzed by Western blot in nuclear (left) and cytosolic (right) extracts. A representative Western blot is shown above, and the densitometric analysis is shown below. (D) Nuclear extracts were harvested from cells incubated with BGP, and then, nuclear extracts were incubated with biotin‐labeled oligonucleotide containing the AP‐1 consensus sequence from ECE‐1 gene. A representative EMSA is shown on the left with a competition assay using nuclear extracts from control cells; lane one, negative control without nuclear extract; lane two, biotin‐labeled AP‐1; lane three, an excess of unlabeled AP‐1; and lane four, an excess of unlabeled NF‐kB. The densitometric analysis of EMSAs is given on the right. Values are the mean ± SEM of six independent experiments, *P < 0.05 vs. ECE‐1 (A) or time zero (B,C), and they are expressed as fold induction between BGP and control cells (A), or as a percentage of control values (B,C). Statistical test applied was one‐way ANOVA with repeated measures, followed by Bonferroni multiple comparison test (A), or followed by Dunnett's multiple comparison test (C). A two‐way RM ANOVA, followed by Bonferroni post‐test, was applied in B.

To study the mechanism involved in the effect of BGP on ECE‐1 expression, several experiments were assessed. First of all, cells were transfected with serial deletions of ECE‐1 promoter and luciferase assays were performed to evaluate the potential transcription factor involved. When the sequence for AP‐1 was deleted in the first deletion (Fig.  2A), BGP effect disappeared compared to the effect over the whole promoter (ECE‐1, Fig.  2A), and remained blocked when the rest of serial deletions of ECE‐1 were tested in the presence of BGP. Secondly, we evaluated whether AP‐1 was actually involved. For this purpose, we studied subcellular localization of c‐Fos and c‐Jun, which are subunits of AP‐1, and we found that both of them go through nucleus between 15 and 120 min (Fig.  2B) after BGP addition, suggesting that they were activated by BGP. Finally, EMSAs were realized to confirm AP‐1 involvement, showing that BGP increased the binding of AP‐1 to nuclear extracts between 30 and 120 min (Fig.  2C).

### Hyperphosphatemia induces senescence in human endothelial cells through ET‐1

**Figure 3:**
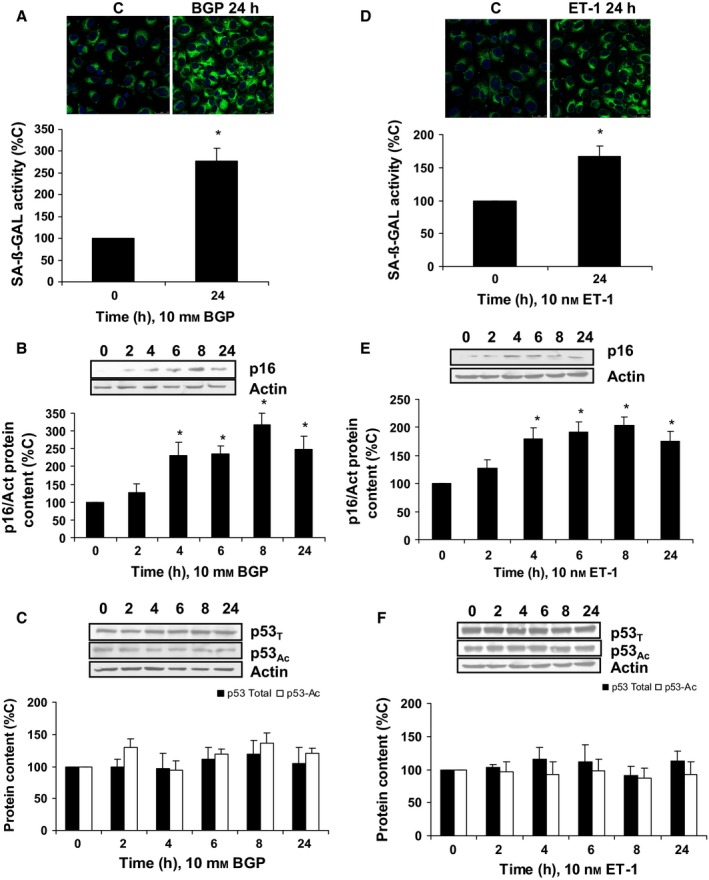
Hyperphosphatemia and ET‐1 induce senescence in human endothelial cells. Cells were incubated with 10 mm β‐glycerophosphate (BGP) (A,B,C) or 10 nm ET‐1 (D,E,F) at indicated times. Senescence was tested measuring SA‐ß‐GAL activity (A,D), representative microphotographs are shown above with 40 × magnification and the densitometric analysis is shown below. Scale bar, 50 μm. Protein content from p16 (B,E) and p53 (C,F) was analyzed. A representative Western blot is shown above, and the densitometric analysis is shown below. Values are the mean ± SEM of six independent experiments, *P < 0.05 vs. control cells (time zero). Statistical test applied was one‐way ANOVA with repeated measures, followed by Bonferroni multiple comparison test.

**Figure 4:**
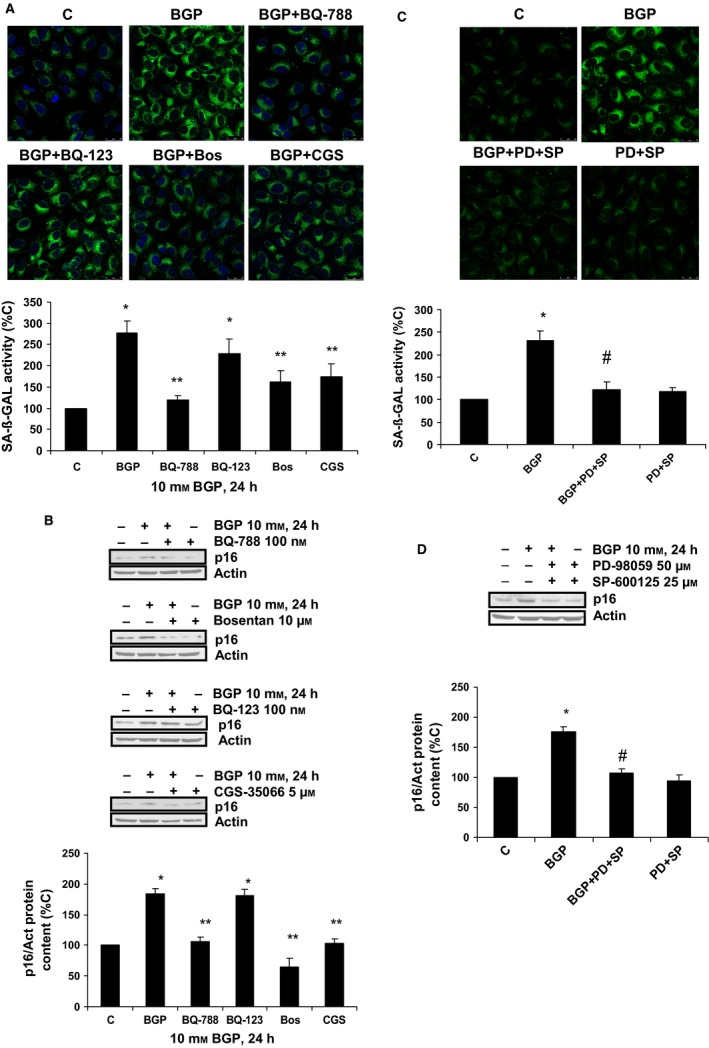
Role of ET‐1 and AP‐1 in hyperphosphatemia‐dependent endothelial senescence: effect of different blockage strategies. Cells were grown on coverslips (A,C) and incubated with 100 nm BQ‐788, 100 nm BQ‐123, 10 μm Bosentan, or 5 μm CGS‐35066 (A,B); or with 50 μm PD‐98059 (PD) and 25 μm SP‐600125 (SP) (C,D). These antagonists were added 30 min before 10 mm β‐glycerophosphate (BGP), and then incubated for 24 h. Then, SA‐ß‐GAL activity (A,C) and p16 protein content (B,D) were analyzed. Representative microphotographs are shown with 40 × magnification, and the densitometric analysis is shown below (A,C). Scale bar, 50 μm. A representative Western blot is shown above, and the densitometric analysis is shown below (B,D). Values are the mean ± SEM of six independent experiments, *P < 0.05 vs. C, **P < 0.05 vs. BGP and BGP + BQ123, and ^#^ *P* < 0.05 vs. BGP. Statistical test applied was one‐way ANOVA with repeated measures, followed by Dunnett's multiple comparison test.

Next, we studied the physiological consequences of the up‐regulation of ECE‐1 and ET‐1 by BGP. BGP induced senescence in human endothelial cells, measured as increased SA‐ß‐GAL activity by fluorescence confocal microscopy at 24 h (Fig.  3A), as well as the increment in p16 expression at different hours (Fig.  3B), without changing in total‐p53 or acetyl‐p53 protein expression (Fig.  3C). To exclude a possible role of hyperphosphatemia on cell death, we assess apoptosis by detection of Annexin V expression using a flow cytometer. There was no difference in the percentage of apoptotic cells after BGP treatment for 24 h with respect to control cells (Fig. S1). In order to demonstrate whether the effect of BGP on senescence was due to the induction of ET‐1 synthesis, we checked the effect of ET‐1 on endothelial senescence by itself. ET‐1 was able to increase SA‐ß‐GAL activity at 24 h (Fig.  3D) and p16 expression without changing total‐p53 or acetyl‐p53 protein expressions (Fig.  3E and F, respectively). Finally, we tested the effect of BGP in the presence of different antagonists of ET‐1 system, such as BQ123, the specific antagonist of ET_A_ receptor; BQ788, the specific antagonist of ET_B_ receptor; and Bosentan, an unspecific ET_A/B_ receptor antagonist. Besides, CGS‐35066, a specific inhibitor of ECE‐1 activity, was used to avoid ET‐1 production (Raoch *et al*., 2007). Bosentan and BQ788, but not BQ123, were able to block the increase in both SA‐ß‐GAL activity (Fig.  4A) and p16 protein expression (Fig.  4B). In addition, CGS‐35066 also inhibited the BGP effect on SA‐ß‐GAL activity and p16 protein expression (Fig.  4A and B). These results confirm the role of ET‐1 in endothelial senescence induced by hyperphosphatemia. In addition, when AP‐1 was blocked using pharmacological inhibitors during BGP treatment, PD‐98059 (PD), which inhibits phosphorylation of ERK_1/2_ by MEK and subsequently c‐Fos, and SP‐600125 (SP), which inhibits JNK and prevents c‐Jun activation; we found that they were able to block endothelial senescence induced by BGP, measured as SA‐ß‐GAL activity (Fig.  4C) and p16 protein expression (Fig.  4D).

### Hyperphosphatemia induces endothelial cell senescence through the generation of reactive oxygen species

**Figure 5:**
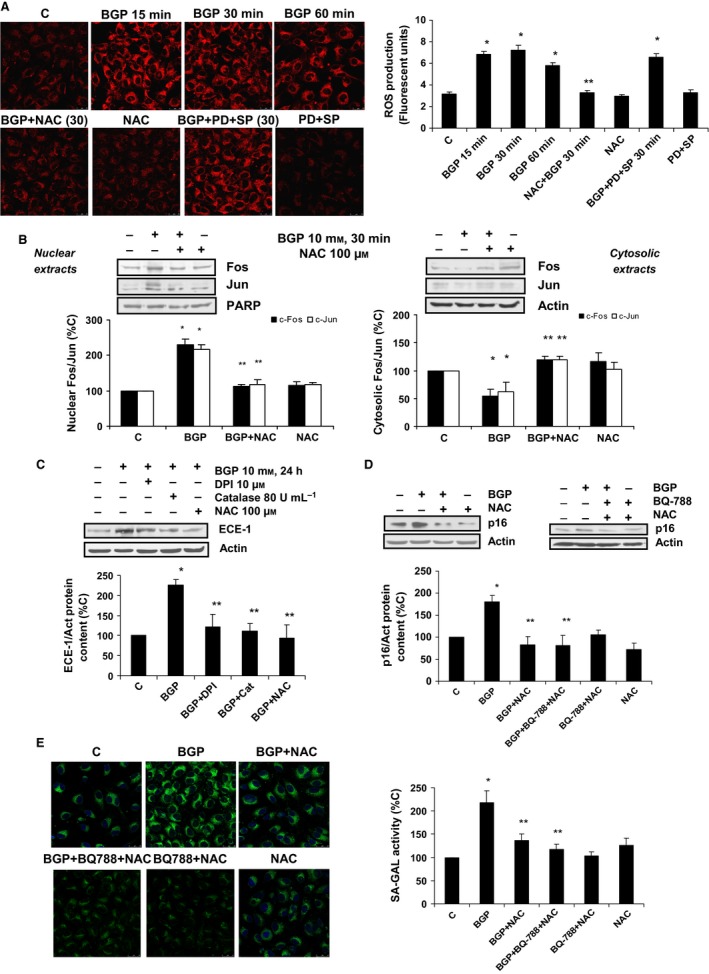
Role of ROS in hyperphosphatemia‐dependent AP‐1 activation, ECE‐1 up‐regulation, and endothelial senescence. (A) Cells were incubated with 10 mm β‐glycerophosphate (BGP) at different times, some cells were incubated in the presence of 100 μm N‐acetyl‐cysteine (NAC) or 50 μm PD‐98059 (PD) plus 25 μm SP‐600125 (SP); or with NAC or PD + SP alone for 30 min. CellROX probe was added during the last 30 min of incubation. After being washed twice, in vivo cells were visualized by microscopy confocal to test ROS production in red. Representative microphotographs are shown on the left with 40 × magnification, scale bar, 50 μm. The densitometric analysis is shown on the right panel A. (B) Cells were incubated with BGP in the presence of 100 μm NAC for 30 min. c‐Fos and c‐Jun protein expressions were analyzed by Western blot in nuclear (left) and cytosolic (right) extracts. A representative Western blot is shown above, and the densitometric analysis is shown below. (C) Cells were incubated with 10 mm BGP in the presence or absence of different antioxidants, 10 μm DPI, 80 U mL^−1^ catalase and 100 μm NAC, which were added 30 min before BGP, and then incubated for 24 h. ECE‐1 protein expression was tested; a representative Western blot was shown above with the densitometric analysis below. (D,E) Cells were grown on coverslips (E) and incubated with BGP in the presence of 100 μm NAC or 100 nm BQ‐788 plus NAC for 24 h. Then, p16 protein content was shown (D), and a representative Western blot was shown above with the densitometric analysis below. SA‐ß‐GAL activity was tested (E), representative microphotographs are shown on the left with 40 × magnification, and the densitometric analysis is shown on the right panel E. Scale bar, 50 μm. Values are the mean ± SEM of six independent experiments, *P < 0.05 vs. control cells and other groups, and **P < 0.05 vs. BGP alone. Statistical test applied was one‐way ANOVA with repeated measures, followed by Dunnett's multiple comparison test (A,C,D). A two‐way RM ANOVA, followed by Bonferroni post‐test, was applied in B.

To explore the intracellular mechanism involved in the effect of hyperphosphatemia on endothelial cells, we tested whether BGP addition increased ROS generation. BGP was able to induce ROS production (Fig.  5A), reaching a maximum around 15‐60 min after addition, and they were completely blocked in the presence of the antioxidant N‐acetylcysteine (NAC) (Fig.  5A). When AP‐1 was blocked using pharmacological inhibitors during BGP treatment, PD and SP, we found that they were not able to block ROS production induced by BGP (Fig.  5A), suggesting that ROS production is first than AP‐1 activation. ROS production seems to be involved in the mechanism of action of BGP, because the use of antioxidants blocked AP‐1 activation at 30 min (Fig.  5B) and then, ECE‐1 expression (Fig.  5C) and endothelial senescence, measured as p16 protein expression (Fig.  5D) and SA‐ß‐GAL activity (Fig.  5E).

### Hyperphosphatemia induces ECE‐1 expression, ET‐1 synthesis, and cellular senescence in vivo

**Figure 6:**
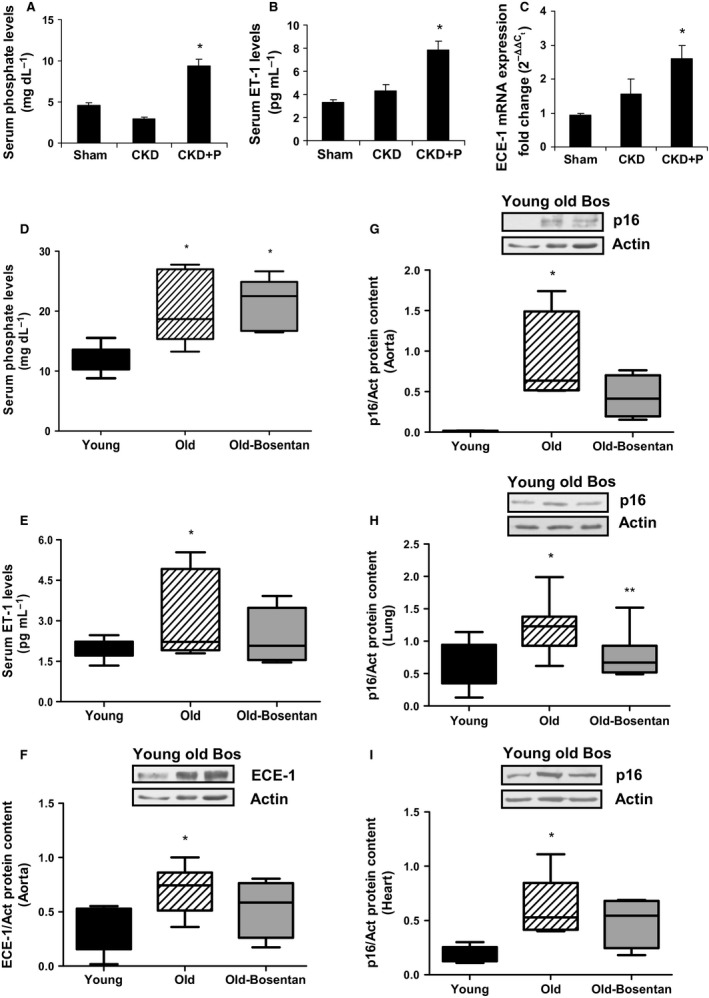
Hyperphosphatemia from animal models resemble the *in vitro* up‐regulation on endothelin system and p16 protein expression. Animals were kept on a 12 h:12 h light–dark cycle, at 24 °C, and food and water were available ad libitum. (A,B,C) Seven per eight nephrectomized rats developed chronic kidney disease (CKD), and then, one group was fed on a normal diet (CKD) and the other on a high phosphate diet (CKD+P) (0.9%) for four weeks, both groups were compared with sham‐operated control rats (Sham). Rats were sacrificed, and blood samples were collected to measure different parameters such as phosphate levels by colorimetric kit (A) and ET‐1 levels by ELISA (B). Aorta tissue was collected to measure ECE‐1 mRNA expression by qPCR using specific TaqMan rat probe (C). Values are the mean ± SEM of ten rats, *P < 0.05 vs. control rats (Sham). The statistical test applied was one‐way ANOVA followed by Dunnett's multiple comparison test. (D,E,F**)** Mice of different ages were used to study physiological aging: 5 month (Young: black bars), 20‐month‐old (Old: stripped bars), and 20‐month‐old‐Bosentan (Old‐Bosentan: gray bars), which were treated with 30 mg kg^−1^ day^−1^ Bosentan administered in drinking water. Mice were sacrificed, and blood samples were collected to measure phosphate levels by colorimetric kit (D) and ET‐1 levels by ELISA (E). Aorta tissue was collected to measure ECE‐1 protein content by Western blot (F). A representative Western blot was shown above with the densitometric analysis below. Aorta (G), lung (H), and heart (I) were collected to assess senescence measured as p16 protein expression. A representative Western blot was shown above with the densitometric analysis below. Values are the mean ± SEM of ten mice, *P < 0.05 vs. young mice, **P < 0.05 vs. old mice. The statistical test applied was one‐way ANOVA followed by Dunnett's multiple comparison test.

To analyze the *in vivo* relevance of the previous results, we chose two different experimental models in which hyperphosphatemia was a common phenomenon, a model of CKD in rats and aged mice. CKD was induced in rats by seven/eight nephrectomy. After surgery, rats were fed on a normal diet or a high phosphate diet (0.9%) for four weeks, and then, they were compared with sham‐operated rats (control rats). CKD rats showed higher levels of urea and creatinine than control rats (data not shown). Serum phosphate and ET‐1 levels were significantly higher in CKD rats fed on a high phosphate diet for 4 weeks compared to CKD rats fed on a normal diet or sham‐operated rats (Fig.  6A and B), without changing in calcium levels (data not shown). In addition, that group showed an up‐regulation in ECE‐1 mRNA expression in aorta tissue (Fig.  6C). In another experimental approach, 5‐month‐old mice were compared with 20‐month‐old mice treated or not with Bosentan administered for the last 8 weeks before sacrifice. Bosentan is a dual antagonist of endothelin receptor that can be administered dissolved in drinking water, to assess the relevance of ET‐1 in the hyperphosphatemia found in old mice. Results showed that older mice had higher levels of phosphate and ET‐1 in serum in relation to the younger mice (Fig.  6D and E). Furthermore, protein expression of ECE‐1 was significantly increased in aorta from older mice (Fig.  6F). Old mice treated with Bosentan did not reduce phosphate levels respect to old mice without treatment (6D). However, Bosentan were able to reduce ET‐1 levels (Fig.  6E) and ECE‐1 protein expression in different cardiovascular tissues such as aorta (Fig.  6F), heart, and lung (Fig. S2). Reduction in ECE‐1 protein expression was only significant in heart tissue. These findings demonstrated that high serum phosphate concentration coexisted with high circulating ET‐1 and high ECE‐1 endothelial expression in both CKD and the aging process.

To confirm the relevance of hyperphosphatemia in senescence from mice, p16 protein expression was measured in different tissues from young and old mice. p16 expression was higher in all tissues analyzed in the older mice (Fig.  6G, H and I). Treatment with Bosentan was able to reduce p16 expression in all tissues, reaching significant in lung tissue (Fig.  6H). These results suggest the potential role of ET‐1 in *in vivo* senescence.

## Discussion

Hyperphosphatemia occurs when the homeostasis of phosphate is altered, as described in patients with CKD and in the premature aging syndromes presented in the Klotho mice and FGF23 KO mice (Razzaque *et al*., 2006; Nakatani *et al*., 2009). The increment in the serum phosphate concentration dramatically affects the behavior of cells resident in the vasculature and has been recognized as a risk factor for the development of cardiovascular events (Lau *et al*., 2001) and mortality. Many works have described the effects of hyperphosphatemia on the vascular smooth muscle cells: induction of osteogenic transformation and vascular calcification (Lau *et al*., 2001) and cellular cycle disruption, which leads to apoptosis and cell senescence (Troyano *et al*., 2015). However, the information about the effect of hyperphosphatemia on endothelial cells is limited to a few works. Recently, it has been described that hyperphosphatemia induces endothelial cell apoptosis (Qin *et al*., 2015) by up‐regulating bone morphogenetic protein‐4, and it also induces the formation of endothelial microparticles with pro‐coagulant properties, which could contribute to occlusive acute events (Abbasian *et al*., 2015). On the other hand, sevelamer, a phosphate scavenger, improved endothelial function and reduced mortality in patients with CKD (Chennasamudram *et al*., 2013; Rastogi, 2013). Hyperphosphatemia induces endothelial dysfunction via various mechanisms, including a decline in nitric oxide release due to oxidative stress (Peng *et al*., 2011). Even transient postprandial hyperphosphatemia may promote endothelial dysfunction (Shuto *et al*., 2009). Our results indicate that vascular dysfunction under hyperphosphatemic conditions could be due to an excess of endothelin‐1 production by endothelial cells as a result of the increased expression of ECE‐1. We used BGP to augment the extracellular phosphate concentration in culture media as previously reported (Troyano *et al*., 2015). Endothelial cells treated with BGP showed an increase in the ECE‐1 expression due to the activation of the AP‐1 transcription factor. For the first time, a direct activation of AP‐1 activity by hyperphosphatemia has been reported.

We demonstrate that endothelial cell senescence promotion is a consequence of increased ET‐1 production after high extracellular phosphate exposition. Senescence can be promoted prematurely in response to stressful stimuli that result in DNA damage (Wang *et al*., 2009), and recent reports from our group have shown that there exists a relationship between hyperphosphatemia and senescent or aging cells (Troyano *et al*., 2015). Cell senescence was analyzed by p16 protein expression and SA‐β‐GAL activity as previously described (Dimri *et al*., 1995), without changing in p53 protein expression. This result indicates that hyperphosphatemia induced the cell cycle arrest leading endothelial cells to senescence and not to apoptosis at the time tested (Terzi *et al*., 2016). (Di Marco *et al*., 2007) found that 2.5 mm inorganic phosphate induced endothelial cell apoptosis *in vitro*. In contrast, we did not find any difference in the percentage of apoptotic cells after BGP treatment for 24 h with respect to control when measuring Annexin V expression, although our experimental conditions were completely different to Di Marco ones. In any case, senescent cells are undergoing to a sure cell death which, probably, will happen later.

Present results suggest that ET‐1 seems to be the main responsible for senescence induced by BGP, at least in endothelial cells. The ET‐1 role in senescence was confirmed because the use of a specific ET_B_ receptor antagonist (BQ788), or the unspecific dual antagonist of ET_A/B_ receptor (Bosentan), was able to block BGP effect significantly, whereas the use of a specific ET_A_ receptor antagonist (BQ123) did not modify BGP effect on senescence. Furthermore, we also used a specific antagonist of ECE‐1 activity (CGS‐35066) to inhibit ET‐1 synthesis, and we found a blockade on BGP effect. In summary, these results confirmed that ET‐1 induced by BGP was able to produce endothelial senescence, just as did ET‐1 itself. This finding can be crucial in vascular function, because senescence induced by ET‐1 at vascular level could be involved in some pathologies (Burton & Krizhanovsky, 2014). In this way, a role for endothelial cell senescence in vascular dysfunction has been described in the context of diabetes (Li *et al*., 2017), varicose patients (Mikuła‐Pietrasik *et al*., 2016), and thrombosis (Bochenek *et al*., 2016).

Elevated circulating level of ET‐1 has been reported in aged humans and activation of endothelin‐1 system has been related to aging‐associated diseases, such as diabetes, atherosclerosis, hypertension, and cancer (Barton, 2014). Also, experimental models of premature aging, such as the telomerase‐deficient mice, have shown increased circulating ET‐1 as a result of the increment in ECE‐1 expression (Pérez‐Rivero *et al*., 2006). ET‐1 up‐regulation has been described in senescent cardiac fibroblast and related to aging‐associated cardiac fibrosis (Wang *et al*., 2015). However, in this work the hypothesis that ET‐1 can induce fibroblast senescence has not been tested.

As in other aging‐related conditions (Chen & Ames, 1994; Macip *et al*., 2002; Radisky *et al*., 2005; Lu & Finkel, 2008), higher levels of ROS seem to be involved in hyperphosphatemia‐induced endothelial cell senescence, which is in accordance with our results. Similarly, other previous results from our group confirmed that ROS were able to up‐regulate ECE‐1 gene (López‐Ongil *et al*., 2002; Martínez‐Miguel *et al*., 2009). In this work, we show that BGP induced ECE‐1 expression and that ROS seemed to be involved because the effect disappeared in the presence of several antioxidants such as DPI, which inhibits NOX, catalase, which inhibits hydrogen peroxide production, and NAC, which inhibits hydrogen peroxide and superoxide anion production. Oxidative stress is involved in many diseases, playing a relevant role in aging.

It is the first time that the potential role of ET‐1 in senescence regulation has been shown. BGP produces ROS, which activates AP‐1, involved in the up‐regulation of ECE‐1, increasing the amount of ET‐1. Then, not only BGP but also ET‐1 itself are able to induce endothelial senescence. Results confirmed that ROS activation was previous to AP‐1 activation, because NAC blocked not only AP‐1 activation, but also ECE‐1 expression and endothelial senescence. Besides, the inhibition of AP‐1 with two pharmacological antagonists, PD‐98059 and SP‐600125, showed a blockage of endothelial senescence but not reduced ROS production.

Lastly, physiological relevance is shown in two experimental animal models; rats with CKD induced by seven/eight nephrectomy and aging mice. We chose those models because patients with CKD usually present high levels of serum phosphate linked to several cardiovascular complications and show premature aging. We tested ET‐1 and phosphate serum levels and the ECE‐1 expression in aorta. Both of them presented high phosphate serum levels as well as higher ET‐1 levels compared with control animals, rats without CKD, or young mice, respectively. Interestingly, rats with CKD fed on a high phosphate diet showed higher ECE‐1 expression in aorta tissue compared with rats with CKD fed on a normal diet or control rats. Similar results were found in aorta from aging mice compared to young mice. Although we did not assess p16 expression in CKD rat model, previous results from our group showed how p16 was increased in nephrectomized rats fed on a high phosphate diet (Troyano *et al*., 2015). Old mice expressed higher levels of p16 protein expression than young mice. However, Bosentan tended to reduce serum ET‐1 levels, ECE‐1 expression and also p16 expression in aorta, lung, and heart tissues. Although these reductions were not always significant, and depended on tissue, in general, confirm the relevance of ET‐1 in senescence process. More experiments are needed to consider Bosentan as a potential therapy. These findings confirm the results found in endothelial cells and suggest the potential role of phosphate during aging or age‐related diseases, suggesting the relationship between aging, hyperphosphatemia, and ET‐1.

In summary, we demonstrated that high levels of phosphate‐induced senescence in cultured endothelial cells through the activation of ECE‐1 and ET‐1 synthesis mediated by ROS production. Also, we provided solid evidences for the in vivo relevance of these results as rats fed on a high phosphate diet and aged mice showed high levels of serum phosphate and ET‐1, as well as more expression of ECE‐1 in aorta tissues. The hyperphosphatemia related to aging or aged diseases could increase senescent cells, which could be involved in impairing or developing other pathologies.

## Experimental procedures

### Materials

Culture plates, culture media, blueStar‐prestained protein marker, BCA protein assay reagent, nitrocellulose membrane, secondary horseradish peroxidase‐conjugated goat anti‐mouse IgG, and CL‐Xposure films were from Cultek (Thermo Fisher Scientific brand, Madrid, Spain), and Supersignal West Pico detection system and LightShift Chemiluminescent EMSA kit were from Pierce (Thermo Fisher Scientific brand, Madrid, Spain). The ET‐1 ELISA system was from Immuno‐Biological Laboratories (IBL Co., Japan). Rabbit polyclonal anti‐c‐Fos and anti‐c‐Jun antibodies were from Santa Cruz BioTechnology (Sta. Cruz, CA, USA); rabbit polyclonal acetyl‐p53 (Lys382) antibody was from Cell Signaling (IZASA, Barcelona, Spain) and rabbit monoclonal anti‐p16 antibody was from Abcam (Cambridge, UK). Acrylamide–bisacrylamide was from Hispanlab‐Pronadisa (Madrid, Spain). Electrophoresis equipment was from Bio‐Rad Laboratories (Richmond, CA, USA). The fluorogenic ImaGene green C_12_FDG substrate reagent and CellROX deep red probe for oxidative stress detection were from Molecular Probes (Thermo Fisher Scientific brand, Madrid, Spain). TRIzol reagent was from Ambion‐Life technologies (Thermo Fisher Scientific brand, Madrid, Spain). Protease inhibitor cocktail tablets and FastStart universal probe master were from Roche Diagnostics S.L. (Barcelona, Spain). QuantiChrom phosphate assay kit (DIPI‐500) was from BioAssay Systems (Hayward, CA, USA). FITC Annexin V apoptosis detection kit was from BD Biosciences Europe (Madrid, Spain). High‐capacity cDNA reverse transcription kit and TaqMan gene expression assays from human or rat were purchased from Applied Biosystems (Thermo Fisher Scientific brand, Madrid, Spain). Canfast transfection reagent was from MolBioLab (Canvax, Madrid, Spain). Dual‐luciferase reporter assay system, pGL3 vector, and pRL‐SV40 vector were from Promega (Walkersville, MD, USA). Human ET‐1, BGP, antagonists from endothelin receptors such as BQ123 for ET_A_, BQ788 for ET_B_, and Bosentan hydrate for ET_A_ and ET_B_, as well as the rest of drugs, antibodies, and reagents (unless otherwise indicated) were from Sigma‐Aldrich‐Fluka Chemical Co. (St. Louis, MO, USA).

### Cell culture

EA.hy926 (EA), a human endothelial cell line, was from American Type Culture Collection (Manassas, VA, USA). Cells were grown in Dulbecco's Modified Eagle Media (DMEM) containing 4.5 g L^−1^ glucose and supplemented with 10% fetal bovine serum, 100 U mL^−1^ penicillin, and 100 μg mL^−1^ streptomycin in an atmosphere of 95% air and 5% CO_2_. Supernatants were collected to measure ET‐1 by ELISA (López‐Ongil *et al*., 2000; Martínez‐Miguel *et al*., 2017).

### Animal studies

Wistar rats were housed in a pathogen‐free temperature‐controlled room (22 ± 2 °C). Aortic tissue samples from 4‐month‐old Wistar rats with chronic renal failure were used. Chronic renal failure was induced in Wistar rats by surgical seven/eight nephrectomy. Then, nephrectomized rats were divided into two groups: one group was fed on a normal diet (CKD: 0.6% phosphorus, 0.6% calcium, and 20% protein content), and the other group was fed on a high phosphate diet (CKD + P: 0.9% phosphorus, 0.6% calcium, and 20% protein content) (Panlab, Barcelona, Spain). Control group were sham‐operated rats fed on a normal diet (Sham). The rats were housed in wire cages and received a diet and water ad libitum for four weeks. At time of sacrifice, rats were housed in metabolic cages for 24 h urine collection. Then, rats were anesthetized, and blood samples were collected by heart puncture exsanguinations. Aorta was obtained and conserved in RNA later solution for mRNA extraction. Serum phosphate, calcium, urea, and ET‐1 were measured by a commercial kit.

Five‐ and twenty‐month‐old male C57BL6 mice were obtained from Janvier Laboratories. Animals were kept on a 12 h:12 h light–dark cycle, at 24 °C, and food and water were available ad libitum. Old mice were divided into groups of ten animals each one, a control group without intervention and Bosentan group. Bosentan (30 mg kg^−1^ day^−1^) (de Frutos *et al*., 2011), a dual endothelin receptor antagonist, was administered in the drinking water for the last 8 wks before sacrifice. Then, mice were anesthetized, and blood samples were collected by heart puncture exsanguinations. Aorta was obtained and conserved in RNA later solution for protein extraction. Serum phosphate and ET‐1 were measured by a commercial kit.

The study design and the experimental protocols were performed in agreement with the Guide for the Care and Use of Laboratory Animals published by the US National Institute of Health (NIH Publication No. 85‐23, revised 1996) and with the European Union regulations (EU Directive 2010/63/EU). The study was revised and approved in accordance with the Ethics Committee from Alcala University for mice (Madrid, Spain) and from Oviedo Research Unit for rats (Oviedo, Spain).

### Measurement of ET‐1 levels

Supernatants of confluent monolayer of EA cells treated for different times with 10 mm BGP were collected, lyophilized, and stored at −70 °C until assay. ET‐1 levels from supernatants of cells and plasma from mice or rats were measured by enzyme‐linked immunosorbent assay (ET‐1 ELISA) according to the kit instructions (IBL Co.) using a 96‐well microtiter plate reader. To generate a standard curve for ET‐1, serial dilutions of ET‐1 ranging from 0.78 to 100 pg mL^−1^ were used. A curve was fitted to the standards, and unknown values were interpolated from the standard curve automatically. The cross‐reactivity of the ELISA ET‐1 antibody, as determined by the concentration giving 50% B/Bmax, was as follows: ET‐1 (100), ET‐2 (3.3), ET‐3 (< 0.1), Big‐ET‐1 (< 0.1), and rat Big‐ET‐1 (0.1).

### Western blot assays

Proteins were obtained from cells using the lysis buffer (20 mm Tris‐HCl pH 7.5, 150 mm NaCl, 1 mm EGTA, 1 mm EDTA, 0.1% sodium deoxycholate, 1% Triton X‐100, 10 mm sodium pyrophosphate) containing a protease inhibitor cocktail. The resulting solution was spun at 13 000 rpm for 30 min at 4 °C. Protein concentration was determined with Bio‐Rad protein assay kit. Equal amounts of protein (30 μg protein per lane) from each sample were separated on SDS‐polyacrylamide gels (PAGE) under reducing conditions and transferred onto nitrocellulose membranes. Membranes were blocked with 5% (w/v) nonfat dry milk in Tween Tris‐buffered saline (TTBS) (20 mm Tris‐HCl pH 7.5, 0.9% NaCl, 0.05% Tween 20) for 1 h at room temperature (R/T), and then incubated with different specific antibodies for ECE‐1, p53, acetyl‐p53, or p16 detection. Mouse monoclonal anti‐ECE‐1 antibody (mAb AEC32‐236, provided by Dr. Kohei Shimada, Biological Research Laboratories, Sankyo Co., Ltd. Tokyo, Japan) was incubated for 90 min at R/T (10 μg mL^−1^ in TTBS with 0.05% BSA), mouse monoclonal anti‐p53 for 1 h at R/T (1:3000 dilution in TTBS with 3% BSA), rabbit polyclonal acetyl‐p53 (Lys382) antibody for 2 h at R/T (1:1000 dilution in TTBS with 0.05% BSA) and rabbit monoclonal anti‐p16 antibody for O/N at 4 °C (1:1000 dilution in TTBS with 3% BSA). After washing in TTBS, blots were incubated for 1 h at R/T with horseradish peroxidase‐conjugated goat anti‐mouse IgG (200‐fold diluted for ECE‐1 and p53) or goat anti‐rabbit IgG (10 000‐fold diluted for acetyl‐p53 and p16), as secondary antibodies. The immunoreactive bands were visualized with the SuperSignal West Pico detection system after 30 s of exposure to CL‐Xposure films. Then, blots were reblotted with a rabbit anti‐actin antibody to normalize ECE‐1, p53, acetyl‐p53 or p16 levels.

Similar experiments were performed in cytosolic and nuclear fractions from control and BGP‐stimulated EA at different times. Proteins were separated on 8% SDS‐PAGE and detected by immunoblot with rabbit anti‐c‐Fos and anti‐c‐Jun antibodies, to study the role of AP‐1. Blots were incubated O/N at 4 °C with 1:750 dilution of each antibody in TTBS with 3% BSA. After washing in TTBS, blots were incubated for 1 h at R/T with 1/10 000 horseradish peroxidase‐conjugated goat anti‐rabbit IgG. The immunoreactive bands were visualized with the SuperSignal West Pico detection system after 30 s of exposure to MXB film. Then, blots were reblotted with a rabbit anti‐actin antibody in cytosolic extracts and rabbit anti‐PARP antibody in nuclear extracts, to normalize c‐Fos and c‐Jun levels.

### Quantitative RT–PCR

Total RNA from EA cells or aorta tissue from rats was isolated using TRIzol reagents according to the manufacturer's protocol. cDNA was synthesized using a High‐Capacity cDNA reverse transcription kit (Martínez‐Miguel *et al*., 2009), and ECE‐1, prepro‐endothelin‐1 (ppET‐1), and beta‐actin or GAPDH expression were measured by quantitative PCR (ABI Prism 7 500 Fast Real‐Time PCR System) and analyzed with 7 500 Fast sequence detection software v1.3.1 (Applied Biosystems Inc., Foster City, CA, USA), using TaqMan genes and Double delta Ct method. TaqMan genes: ECE‐1 (Hs01043735_m1, Rn00585943_m1), prepro‐ET‐1 (Hs00174961_m1) and the endogenous control beta‐actin (Hs99999903_m1) and GAPDH (Rn01775763_g1) were used.

### Transient transfection experiments

To determine whether the effect of BGP on ECE‐1 gene expression was mediated by the 5′‐flanking region of the gene, endothelial cells were transfected using the pGL3‐ECE‐1 plasmid containing the complete human ECE‐1 promoter or serial deletion fragments (A to G) linked to luciferase reporter gene, as described (López‐Ongil *et al*., 2002; Martínez‐Miguel *et al*., 2009). EA cells were grown at 60‐80% of confluence in 12‐well plates and transfected with each luciferase construct, by mixing plasmid DNA (0.1 μg μL^−1^ of luciferase reporter, 1 ng μL^−1^ of plasmid control from *Renilla* luciferase, pRL‐SV40 vector and 10 μg mL^−1^ of Canfast into complete DMEM). After 24 h of transfection, cells were incubated with complete DMEM for 24 h, and then, BGP was added in some wells at different times using serum‐free DMEM. Luciferase activity was assessed using a dual‐luciferase reporter assay system and expressed as relative light units of each plasmid DNA per Renilla per mg protein of each well.

### Electrophoretic mobility shift assays (EMSA)

Nuclear extracts from EA treated with BGP at different times and electrophoretic mobility shift assays were displayed to check on the activation of AP‐1, as previously described (López‐Ongil *et al*., 2002; Raoch *et al*., 2007).

To detect DNA–protein interactions, we used the LightShift Chemiluminescent EMSA Kit which uses a nonisotopic method. Oligonucleotide sequences were based on the putative AP‐1 binding element in the ECE‐1 promoter (from nucleotides −640 to 669; 5′‐CCC TGC **ACT TCC TCT C**AT TGT GCC TCC‐3′) (Valdenaire *et al*., 1995). Biotin end‐labeled DNAs containing the binding site of interest (AP‐1 from ECE‐1) were briefly incubated with 1 μg μL^−1^ nuclear extracts. Protein–DNA complexes were subjected to gel electrophoresis on a native polyacrylamide gel in 0.5 × Tris Buffer EDTA and then transferred to a positively charged nylon membrane. The biotin end‐labeled DNA was detected using the streptavidin–horseradish peroxidase conjugate and the chemiluminescent substrate as described in the kit. For competition experiments, 200‐fold molar excess of competitor DNA (NF‐kB oligonucleotides) was co‐incubated with biotin end‐labeled DNAs (biotin‐AP‐1). To analyze the specificity of DNA–protein complex, 200‐fold molar excess of rabbit polyclonal IgG antibodies for anti‐c‐Jun and anti‐c‐Fos was co‐incubated with the biotin‐AP‐1, respectively.

### ROS production

EA cells were grown in 60μ dishes^35 mm, high^ with glass bottom (Ibidi, Martinsried, Munich, Germany), and after 24 h of free‐serum DMEM, cells were treated at different times with BGP in presence or not of the antioxidant N‐acetyl cysteine (NAC). ROS production was measured by fluorescence confocal microscopy, using the CellROX Deep Red probe. After BGP treatment, 5 μm CellROX was added for the last 30 min. At the end of incubation, cells were washed twice with PBS, and fixed with 4% paraformaldehyde for 15 min. Cells were analyzed using LEICA TCS‐SP5 confocal microscope (Leica Microsystems; Wetzlar, Germany) at 633 nm helium–neon laser to detect red fluorescence of CellROX probe. Pictures were obtained, and fluorescence intensity was measured by densitometry by imagej software (http://rsbweb.nih.gov/ij/).

### Detection of senescence‐associated ß‐galactosidase activity by fluorescence confocal microscopy

EA cells were grown in microscope cover glasses, and after 24 h of free‐serum DMEM, they were treated with BGP, at different times, in the presence or not of different antagonist. To determine cellular senescence, SA‐ß‐GAL activity was measured by fluorescence confocal microscopy, using the fluorogenic substrate C_12_FDG (Dimri *et al*., 1995). After BGP treatment, 33 μm C_12_FDG was added for 2 h. At the end of incubation, cells were washed twice with PBS, and fixed with 4% paraformaldehyde for 15 min. Subsequently, cells were washed again and mounting in ProLong^®^ Gold antifade reagent with DAPI overnight. Samples were analyzed using LEICA TCS‐SP5 confocal microscope (Leica Microsystems; GmbH, Mannheim, Germany) at 488 nm argon laser to detect green fluorescence of SA‐ß‐GAL activity and at 405 nm to detect DAPI. Pictures were obtained, and fluorescence intensity was measured by densitometry by imagej software (http://rsbweb.nih.gov/ij/).

### Statistical analysis

graphpad Prism five software was used for statistical analysis. Statistical tests employed are described in figure legends. Unless otherwise specified, data are expressed as the means ± SEM, and as a percentage of the control values. The level of statistically significance was defined as *P* < 0.05.

## Funding

This work was supported by grants from the Fondo de Investigaciones Sanitarias from Instituto de Salud Carlos III (grants: PI13/02270; PI13/00336; PI13/00014; PI16/02082; PI16/01619), Networks Program REDinREN from Instituto de Salud Carlos III and FEDER funds (grant RETIC REDinREN: RD12/0021/0006; RD12/0021/1023; RD016/009), and Fundación de Investigación Biomédica del Hospital Príncipe de Asturias (grant: FIB‐PI13‐01). Diana Medrano holds a contract as a technician from Plan Estatal I+D+i de Investigación Científica y Técnica y de Innovación (PTA2012‐7570‐I), and Susana López‐Ongil holds a contract from the Research Stabilization program from Instituto de Salud Carlos III (CES07/032).

## Author contributions

D Rodríguez‐Puyol, L Rodríguez‐Mañas, MP Ruíz‐Torres, and S López‐Ongil designed the study. G Olmos, P Martínez‐Miguel, E Alcalde‐Estevez, D Medrano, and P Sosa have contributed by performing experiments, analyzing data, and preparing figures. M Naves‐Diaz has contributed by providing the data obtained from rats. All authors, except D Medrano, have interpreted results of experiments. G Olmos, MP Ruíz‐Torres, and S López‐Ongil have written a draft article, which has been revised by D Rodríguez‐Puyol, L Rodríguez‐Mañas, and M Naves‐Diaz. All authors have read and approved the final version of the manuscript.

## Conflict of interest

None declared.

## Supporting information


**Fig. S1** Hyperphosphatemia did not induce cell death in human endothelial cells.

**Fig. S2** Aged mice increased ECE‐1 protein content. Mice of different ages were used to study physiological aging: 5 month (Young: black bars), 20‐month‐old (Old: stripped bars) and 20‐month‐old‐Bosentan (Old‐Bosentan: gray bars), which were treated with 30 mg kg^−1^ day^−1^ Bosentan administered in drinking water.
