# Supplementary figures and images for: Hyperphosphatemia induces senescence in human endothelial cells by increasing endothelin‐1 production
Source: Aging Cell. 2017 Aug 31;16(6):1300–12. doi: 10.1111/acel.12664 (PMC5676064; doi:10.1111/acel.12664)

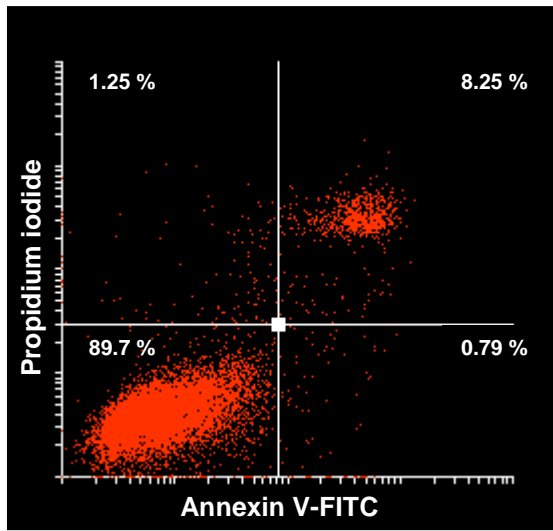

Control

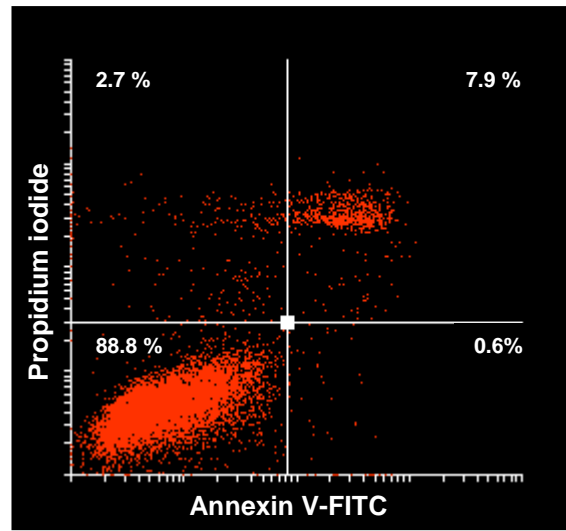

BGP 10 mM, 24h

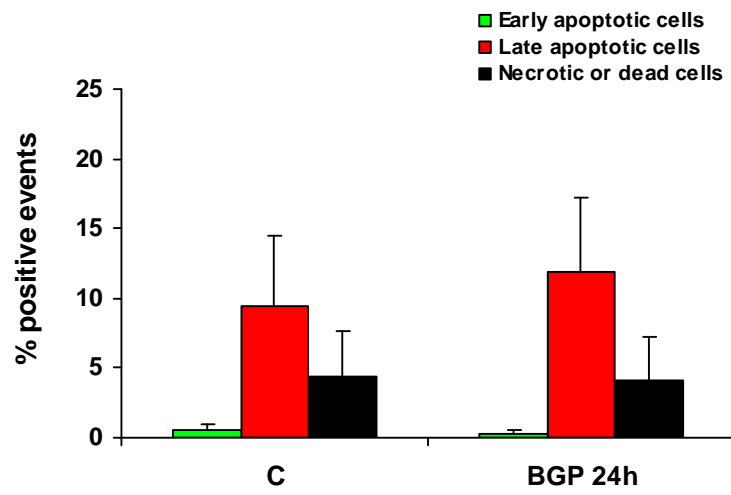

Supplement: Supplementary file 1 — Fig. S1 Hyperphosphatemia did not induce cell death in human endothelial cells. [file ACEL-16-1300-s001.pdf]

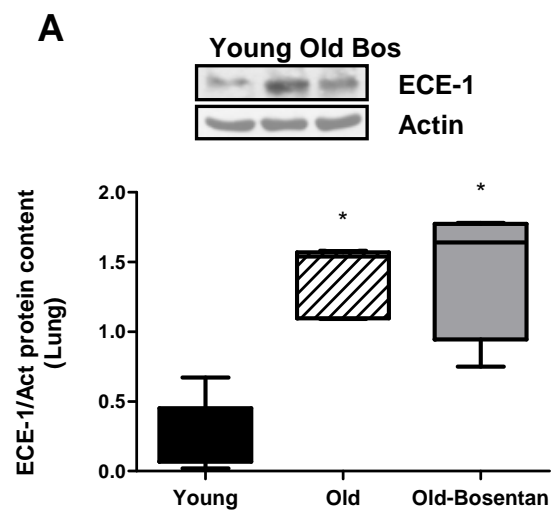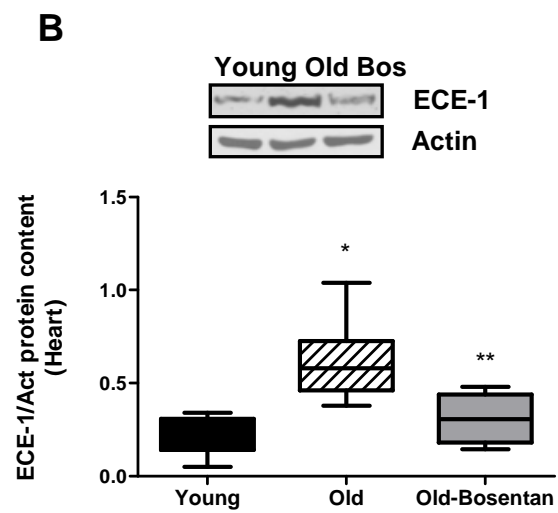

Supplement: Supplementary file 2 — Fig. S2 Aged mice increased ECE‐1 protein content. Mice of different ages were used to study physiological aging: 5 month (Young: black bars), 20‐month‐old (Old: stripped bars) and 20‐month‐old‐Bosentan (Old‐Bosentan: gray bars), which were treated with 30 mg kg−1 day−1 Bosentan administered in drinking water. [file ACEL-16-1300-s002.pdf]
